# Contrasting associations between breeding coloration and parasitism of male Arctic charr relate to parasite species and life cycle stage

**DOI:** 10.1038/s41598-019-47083-x

**Published:** 2019-07-23

**Authors:** I. B. Johansen, E. H. Henriksen, J. C. Shaw, I. Mayer, P.-A. Amundsen, Ø. Øverli

**Affiliations:** 1 Department of Food Safety and Infection Biology, Faculty of Veterinary Medicine, Norwegian University of Life Sciences, P.O. Box 369, Sentrum, N-0102 Oslo Norway; 2 Department of Arctic and Marine Biology, Faculty of Biosciences, Fisheries and Economics, UiT The Arctic University of Norway, P.O. Box 6050, Sentrum, Langnes Tromsø Norway; 3 Marine Science Institute, University of California, Santa Barbara, Santa Barbara, CA 93106-6150 USA; 4 Department of Production Animal Clinical Sciences, Faculty of Veterinary Medicine, Norwegian University of Life Sciences, P.O. Box 369, Sentrum, N-0102 Oslo Norway

**Keywords:** Parasitic infection, Reproductive biology, Behavioural ecology, Freshwater ecology

## Abstract

Conspicuous carotenoid ornamentation is considered a signal of individual “quality” and one of the most intensely studied traits found to co-vary with parasitism. Since it has been suggested that only “high quality” individuals have enough resources to express excessive sexual ornaments and resist parasites, current theory struggles to explain cases where the brightest individuals carry the most parasites. Surprisingly little emphasis has been put on the contrasting routes to fitness utilized by different parasite species inhabiting the same host. Using Arctic charr (*Salvelinus alpinus*) as model species, we hypothesized that skin redness and allocation of carotenoids between skin and muscle (redness ratio) will be positively and negatively associated with parasites using the fish as an intermediate and final host, respectively. Both pigment parameters were indeed positively associated with abundances of parasites awaiting trophic transmission *(Diplostomum* sp. and *Diphyllobothrium* spp.) and negatively associated with the abundance of adult *Eubothrium salvelini* tapeworms. These empirical data demonstrate that contrasting associations between carotenoid coloration and parasite intensities relates to the specific premises of different parasite species and life cycle stages.

## Introduction

Animal coloration has spurred some of the most controversial research in evolutionary biology. Even before Darwin, color signals such as mimicry, warning colors and sexual ornamentation provided pioneering evidence for sexual selection and evolution. Among the most widespread and spectacular ornamental traits in animals are red, orange and yellow carotenoid-based color displays^1–6^, believed to have arisen through strong mating preference for bright colors. Indeed, conspicuous sexual ornamentation is considered a text book example of an “honest” signal of individual “quality” in the sense that carotenoids are in limited supply and incurs a trade-off between use in external signaling versus use in other critical functions in the body (*e*.*g*. immune protection against parasites and pathogen infection).

Indeed, animal pigmentation is one of the most intensely studied phenotypic traits found to co-vary with parasitism^1,4,7–9^. In several species of bird for example, the brightest individuals tend to carry fewer parasites^5,10^. One example is provided by the blackbird (*Turdus merula*), where the individuals with the brightest beaks carry fewer intestinal parasites (*Isospora* Protozoa, Apicomplexa)^5^. Thus, considering the dual use of carotenoids for host physiological responses against parasites and for sexual ornamentation (*i*.*e*. physiological trade-off theory), bright ornamentation can be taken to signal good health and parasite resistance. Under this physiological trade-off framework, however, cases where the brightest individuals are found to carry more parasites than their paler conspecifics are more difficult to reconcile.

In teleost fish with pronounced carotenoid displays, several cases of positive associations between carotenoid-based ornamentation and parasite abundance have been reported^2,6,11^. For example, the Arctic charr (*Salvelinus alpinus* L.) with the brightest red breeding coloration carry more eye flukes (trematode *Diplostomum* spp. metcercariae)^1^. Theories explaining such positive associations are less straightforward, but include the “immunocompetence handicap-principle” suggesting that bright individuals signal that they are of high quality, despite carrying many parasites^1,7,12^. The processes described by this theory have, however, proven difficult to demonstrate empirically^13^. Skarstein and Folstad suggested that higher parasite intensities in more ornamented Arctic charr could be related to immunosuppressive effects of sex hormones necessary for ornament development although sex hormones were not quantified in their study^1^. They also suggested that increased susceptibility to parasites may represent a cost that ensures honesty of carotenoid ornamentation. This theory is, however, complicated by cases where both positive and negative associations between carotenoid coloration and parasite intensities are observed. In three-spined stickleback (*Gasterosteus aculeatus*), for example, the individuals with the brightest red breeding coloration suffer from more muscle-dwelling (*Diphyllobothrium* spp.), but fewer (*Schistocephalus solidus*) plerocercoids^11^.

Thus, whether bright carotenoid ornamentation signals parasite resistance (physiological trade-off theory) or high quality despite increased parasite susceptibility (immunocompetence handicap-principle) is still an open question that begs for further exploration. Moreover, since the two existing theories seem somewhat contradictory, other alternative explanations should be explored. For instance, color polymorphisms may be associated with other phenotypic traits affecting encounter probability or resistance^14^. In this context, we note that little emphasis has been put on the large diversity of parasite species found to co-vary with carotenoid coloration. Indeed, studies investigating carotenoid-based pigmentation in parasitized animals largely focus on host quality and fitness and appear to neglect that parasites also need to heed their own life cycle and fitness.

For example, many helminth parasites (*e*.*g*. nematodes, trematodes, cestodes and acantacephalans) depend on a host being eaten by another host (*i*.*e*. trophic transmission) to complete their life cycle^15–17^. Hence, whereas adult parasites aiming to maximize reproductive output in the gut of their current and final host should benefit from a long-lived host that stays away from danger, trophically transmitted parasites should actually be under selection pressure to make their host more conspicuous or available to the next host in their life cycle^16^. Indeed, many trophically transmitted parasites modify the behavior or appearance of their intermediate host to increase the risk of being preyed upon^15–17^. Lafferty^16^ noted that some parasites may benefit from residing in brighter intermediate hosts. For example, *Diphyllobothrium (spp*.), uses copepods (a crustacean) as first intermediate host, teleosts as second intermediate host, and birds as definitive hosts. Lafferty hypothesized that this parasite may occur more frequently in brighter colored sticklebacks since copepods represent a rich and important source of carotenoids for these fish. Thus, in addition to making male sticklebacks more attractive to females, carotenoids may also make them easier to spot for predatory birds^16^. With this assumption, we would expect a positive relationship for all parasites transmitted to sticklebacks via copepods. That is not always the case. On the contrary, the tapeworm, *S*. *solidus*, that is also transmitted via carotenoid-rich copepods, is actually more abundant in paler sticklebacks^11^.

Thus, proximate mechanisms behind positive and negative associations between parasites and carotenoid displays are probably more complex and could instead involve parasites actively modifying carotenoid allocation to or from the skin. However, whether contrasting reports on associations between parasitism and coloration (*i*.*e*. high parasite load and bright coloration versus high parasite load and pale coloration) can be explained by the circumstance that individual hosts can be infected with multiple parasite species possessing divergent life strategies (*i*.*e*. aiming to maximize reproductive output in final host or ascending the trophic chain to reach its next host) is unknown. In fact, whether parasites benefitting from a more conspicuous host are more abundant in brighter individuals and vice versa has to our knowledge not been tested specifically.

**Figure 1:**
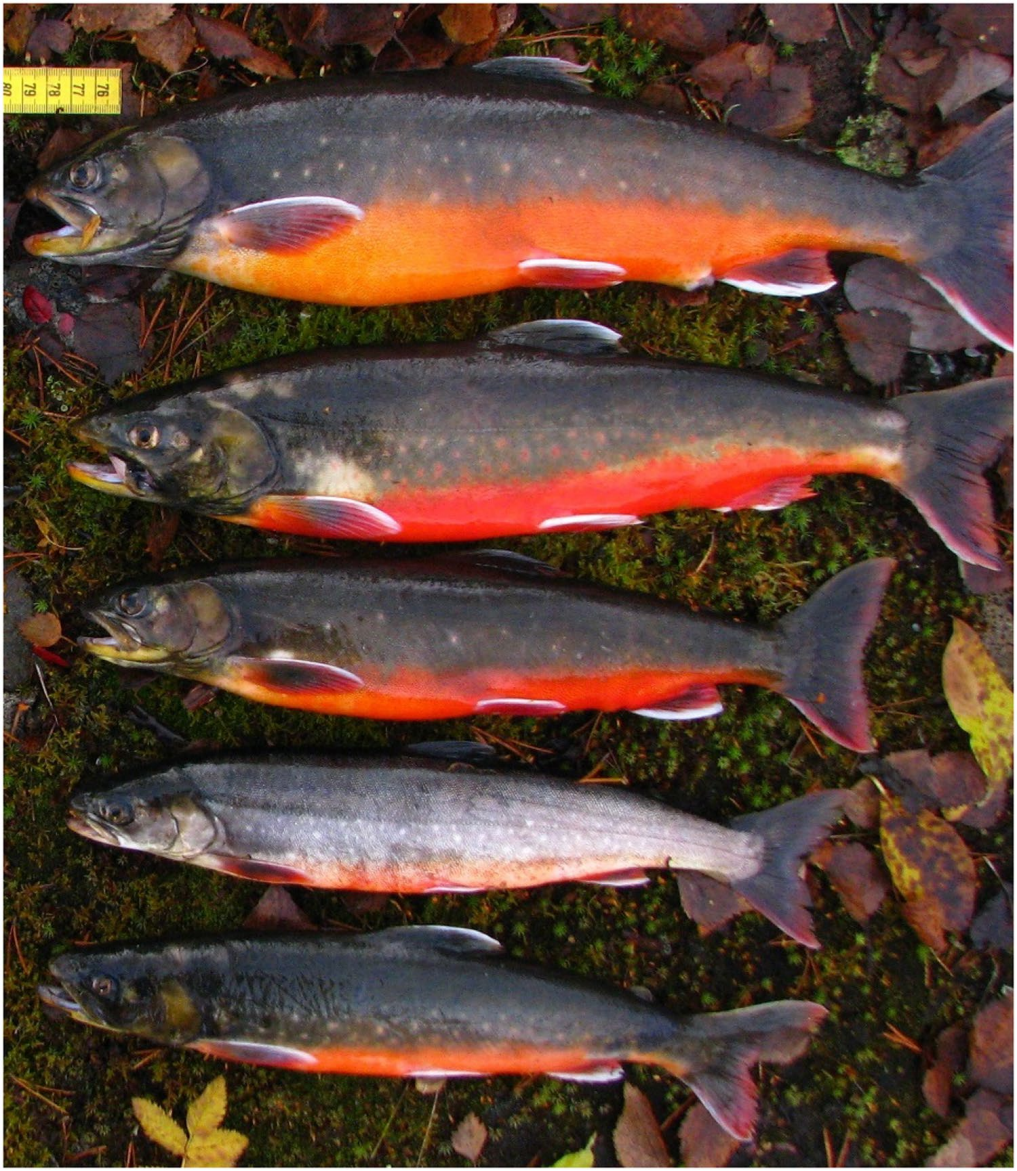
Illustration of variation in individual skin colour in a population of Arctic charr. Photo: Rune Knudsen.

In the present study, we take the parasites’ perspective on conspicuous carotenoid-based ornamentation. To investigate whether parasites benefitting from a more conspicuous host are more abundant in brighter individuals, and vice versa, we used a species that is ideal for studying phenotypic variation in the context of parasites and pigmentation, the Arctic charr. The Arctic charr has been referred to as “the most variable vertebrate on earth”^18^, and one of its striking characteristics that makes it particularly suitable for studying intraspecific variation is that individual skin color varies from light pink to dark red within a given population (see Fig. 1). As mature Arctic charr enter their reproductive season in the autumn, carotenoid pigments are moved from the muscle to the skin, so that the muscle becomes paler in color while the skin becomes more red and conspicuous. This phenomenon allows for observing the coarse distribution between use of pigment for ornamentation in skin versus use in other somatic functions internally. Moreover, Arctic charr inhabiting lakes in northern Norway harbor a diverse community of parasites (Table 1). Some of these parasites (*e*.*g*. *Diplostomum* sp. and *Diphyllobothrium spp*.) are trophically transmitted from charr to bird final hosts^19,20^ and would benefit from a host that is more susceptible to predation. Others (*e*.*g*. *Eubothrium salvelini*, *Cyathocephalus truncatus* and *Protocephalus sp*.) are transmitted from various intermediate hosts to definitive charr hosts where they can live for months to years^21^. Predation or death from other causes associated with high reproductive effort (*e*.*g*. immune deficiency) would not constitute a fitness advantage for such species.

**Table 1:** Parasite species recorded from *Arctic charr* in the present study.

Parasite species	Taxonomic group	1^st^ intermediate host	2^nd^ intermediate host (if present)	Final host	Transmission mode to fish
*Crepidostomum* sp.	Trematoda	Bivalve	Insect/Amphipod	Fish	Trophic
*Cyathocephalus truncatus*	Cestoda	Amphipod		Fish	Trophic
*Cystidicola farionis*	Nematoda	Amphipod		Fish	Trophic
*Diphyllobothrium* spp.	Cestoda	Copepod	Fish	Bird	Trophic
*Diplostomum* sp	Trematoda	Gastropod	Fish	Bird	Direct
*Eubothrium salvelini*	Cestoda	Copepod		Fish	Trophic
*Proteocephalus* sp.	Cestoda	Copepod		Fish	Trophic

Specific objectives of the study were to test the assumption that different parasite species with diverging life history strategies may show contrasting associations with carotenoid pigmentation in male Arctic charr. Also introducing the ratio of carotenoid-based coloration in skin to muscle as an indicator of pigment distribution represents a novel approach that better indicates allocation of pigments from internal stores to external signaling than skin redness *per se*. Specifically, we hypothesize that by quantifying infection intensities of multiple species of parasites in each individual host, it will be demonstrated that (1) multiple and even contrasting associations between parasite load and carotenoid allocation will be discovered and specifically (2) that skin redness and/or skin to muscle redness ratio will be positively associated with intensity of parasites using charr as an intermediate host and negatively associated with intensity of parasites using charr as final host.

## Methods

### Capture and sampling

We sampled Arctic charr from lakes Fjellfrösvatn (69°05′N, 19°20′E), Takvatn (69°07′N, 19°05′E) and Sagelvvatn (69°11′N, 19°06′E) in Troms county, northern Norway. All three lakes are dimictic and oligotrophic, and usually ice-covered from late November to May (see Supporting Table S1 for abiotic characteristics of the three study lakes). Charr in these lakes normally spawn from mid-September to early October^1,22,23^, so we collected fish on their shallow-water spawning grounds between 7–9 September 2016 using gill nets deployed for a maximum of 2 hours. Live fish were removed from the gill nets, placed in buckets containing 0.25 mg l^−1^ MS-222 (Sigma-Aldrich, St. Louis, Missouri, USA) and transported back to land. For each fish we measured fork length (+/− 1.0 mm), determined skin coloration (see Quantification of muscle coloration and parasite intensities section below), and collected a blood sample from the caudal vein using a 1 ml syringe containing ethylendiamin etetraacetic acid (EDTA), after which the fish were humanely killed by decapitation. Blood samples were centrifuged for 5 min at 700 g in a portable mini centrifuge. Plasma was frozen on dry ice and stored at −20 °C for later analysis of Testosterone (T) and 11-keto Testosterone (11kT) levels. Both eyes were dissected from each fish, each eye bagged separately in a small amount of saline solution, and stored on ice for quantification of *Diplostomum* sp metacercariae. The rest of the fish was stored on ice for quantification of parasites (Table 2). We caught only 10 sexually mature female Arctic charr. The strength of pigmentation and their association to parasite infection differ between Arctic charr sexes^1,23^, and we therefore excluded females from our analyses. This study was performed in strict accordance with the Norwegian legislation. For the fish sampling, a fishing permission is required from the fishing right owner. Accordingly, we obtained permissions for the gill net fishing in the lakes Takvatn, Fjellfrösvatn and Sagelvvatn from the County Authority of Troms County (permission reference number: 16/1356-9) with legal authority through LOV 1992-05-15 nr 47, §13. No ethical permission is required from the Norwegian Animal Research Authority for the sampling and described activities (FOR 1996-01-15 no 23, the Norwegian Ministry of Agriculture and Food).

**Table 2:** Estimated fixed effects (including intercept) predicting skin redness after model selection based on AIC values. Marginal *R*^2^ = 0.49, conditional *R*^2^ = 0.49.

Predictor variable	Coefficient (SE)	df	*t* value	*P* value
Intercept	52.55 (1.40)	62.00	37.60	<0.001
*C*. *farionis*	4.53 (2.34)	62.00	1.94	0.058
*Diplostomum* sp.	4.97 (1.49)	62.00	3.33	0.001
*E*. *salvelini*	−5.74 (1.62)	62.00	−3.55	<0.001
11kT	3.40 (1.41)	62.00	2.41	0.019
Fish length	4.59 (1.94)	62.00	2.36	0.021
*C*. *farionis*: *Diplostomum*	6.31 (3.51)	62.00	1.80	0.077
*C*. *farionis*: *E*. *salvelini*	3.27 (1.03)	62.00	3.16	0.002
*Diplostomum: E*. *salvelini*	4.07 (2.61)	62.00	1.56	0.124

### Quantification of parasite intensities

An overview of the parasite species found and their life cycles are given in Table 1. The number of *Diphyllobothrium* sp. cysts on the stomach wall was counted. There are two species of *Diphyllobothrium* present in charr from these systems, *D*. *dendriticum* and *D*. *ditremum*, and cyst counts provide a reliable estimate of their combined total number^24^. Intestines were frozen and later screened for parasites as described by Kuhn *et al*., (2017). Four metazoan parasites were identified from the intestines: *Crepidostomum* sp., *Cyathocephalus truncatus*, *Eubothrium salvelini* and *Proteocephalus* sp. We dissected the eyes and counted the number of *Diplostomum* sp. metacercariae within 24 hours of removal from the fish (with the exception of one individual counted after 48 hours). Finally we counted the total number of *Cystidicola farionis* nematodes from swimbladders preserved in 96% ethanol.

### Determination of carotenoid coloration

To rate skin and muscle coloration, we created a redness scale based on the picture in Fig. 1, a gradient of 11 colors from bright pink to dark red, which yielded a redness score ranging from 0–100 (Fig. S1). If the coloration of skin or muscle was considered to be intermediate between two consecutive colors in the scale, we used the average score between the two colors. Skin coloration was determined for the abdominal area. If skin coloration varied considerably across the abdominal area, an average score was given (for example see Supplementary Fig. S2). Muscle coloration was determined in an area of the muscle located dorsolaterally. The ratio between skin and muscle coloration (redness ratio) was determined by dividing the redness score for the skin by the redness score for the muscle. To estimate reproducibility of color determination using the color scale, the average correlation between 8 different observers using the scale to score skin color of 8 fish was assessed. Average correlation was 94% (linear regression, average ± s.e.m. r^2^ = 0.94 ± 0.05).

### Steroid measurement

Plasma concentrations of T and 11kT (ng/ml) were measured by means of radioimmunoassay (RIA) according to the method described by Frantzen *et al*.^25^. Briefly, steroids were first extracted from plasma samples using diethyl ether and then resolubilized in 300 µl cold RIA buffer. Samples were assayed in duplicate, together with 200 µl antiserum (gift from Dr. Helge Tveiten, University of Tromsø) and 50 µl ^3^H-labelled T or 11kT (Perkin-Elmer, USA). The tubes were vortexed and incubated overnight at 4 °C, after which 300 µl cold dextran-coated charcoal solution was added to all tubes, incubated for 5 minutes and then centrifuged for 5 minutes at 4000 rpm. The supernatant was decanted into scintillation tubes containing 4 ml scintillation fluid (Ultima Gold, Perkin-Elmer, USA) and counted in a liquid scintillation counter (Packard, TRI-CARB 2100TR). The cross-reactivity of the T and 11kT antisera is given by Frantzen *et al*.^25^. Blood sampling failed for two individuals, and these were treated as missing values in the statistical analysis.

### Statistical analysis

For statistical analyses R v. 3.5.1. was used^26^. To test whether pigmentation was predicted by parasite infection we used two separate linear mixed models with skin redness and redness ratio as the respective response variables using the ‘lmer’ function from the ‘lmerTest’ package in R^27^. Initial fixed effects included the abundance of individual parasite species (listed in Table 1), their two-way interactions as well as fish length, T and 11-kT. Fixed effects were standardized by subtracting the mean and dividing by the standard deviation for each data point. Lake was included as a random effect to account for potential variation between populations. Models were stepwise simplified based on AIC values using the ‘dregde’ function from the ‘MuMIn’ library in R^26^. We compared models with random slope and intercept to random intercept only models to see if pigmentation and parasitism associated differently between populations. Random intercept only models performed better (lower AIC value) and are thus presented here. We assessed variance homogeneity and normal distribution of the residuals from diagnostic plots to ensure that we did not violate model assumptions. One fish from Fjellfrösvatn with extreme redness ratio was removed following inspection of model diagnostic plots. The predicted association between fixed effects and pigmentation by the model were plotted using the ‘Effect’ function from the ‘effects’ library in R^28^.

## Results

### Parasite infections

A total of 62 sexually mature males were caught on the spawning grounds across the three study lakes. There were some differences in parasite community composition between lakes. All fish were infected with *Diphyllobothrium* spp. and *Diplostomum* sp. (Table S2). *Crepidostomum* sp., *C*. *farionis* and *E*. *salvelini* were common in Takvatn and Fjellfrösvatn, whereas charr from Sagelvvatn had high infections of *Proteocephalus* sp. (Table S2). *Cyathocephalus truncatus* was common only in Fjellfrösvatn (62% prevalence) at a low abundance. Within lakes, individual charr varied considerably in levels of parasitic infection.

### Coloration

There was an inverse relationship between skin and muscle redness (Pearson’s *r* = −0.30, *P* = 0.02). There were no significant differences in skin redness between lakes (one-way ANOVA, F_60, 2_ = 1.11, *P* = 0.34), and average redness scored between 50 and 60 for all lakes (Supplementary Table S2). Muscle redness was higher in Sagelvvatn than in the two other lakes (Tukey’s HSD test, both *P* < 0.001), and consequently redness ratios were comparatively lower in Sagelvvatn than in both Fjellfrösvatn (Tukey’s HSD test, *P* = 0.003) and Takvatn (Tukey’s HSD test, *P* < 0.001). There was a positive correlation between skin redness and redness ratios across all fish (Pearson’s *r* = 0.54, *P* < 0.001).

**Figure 2:**
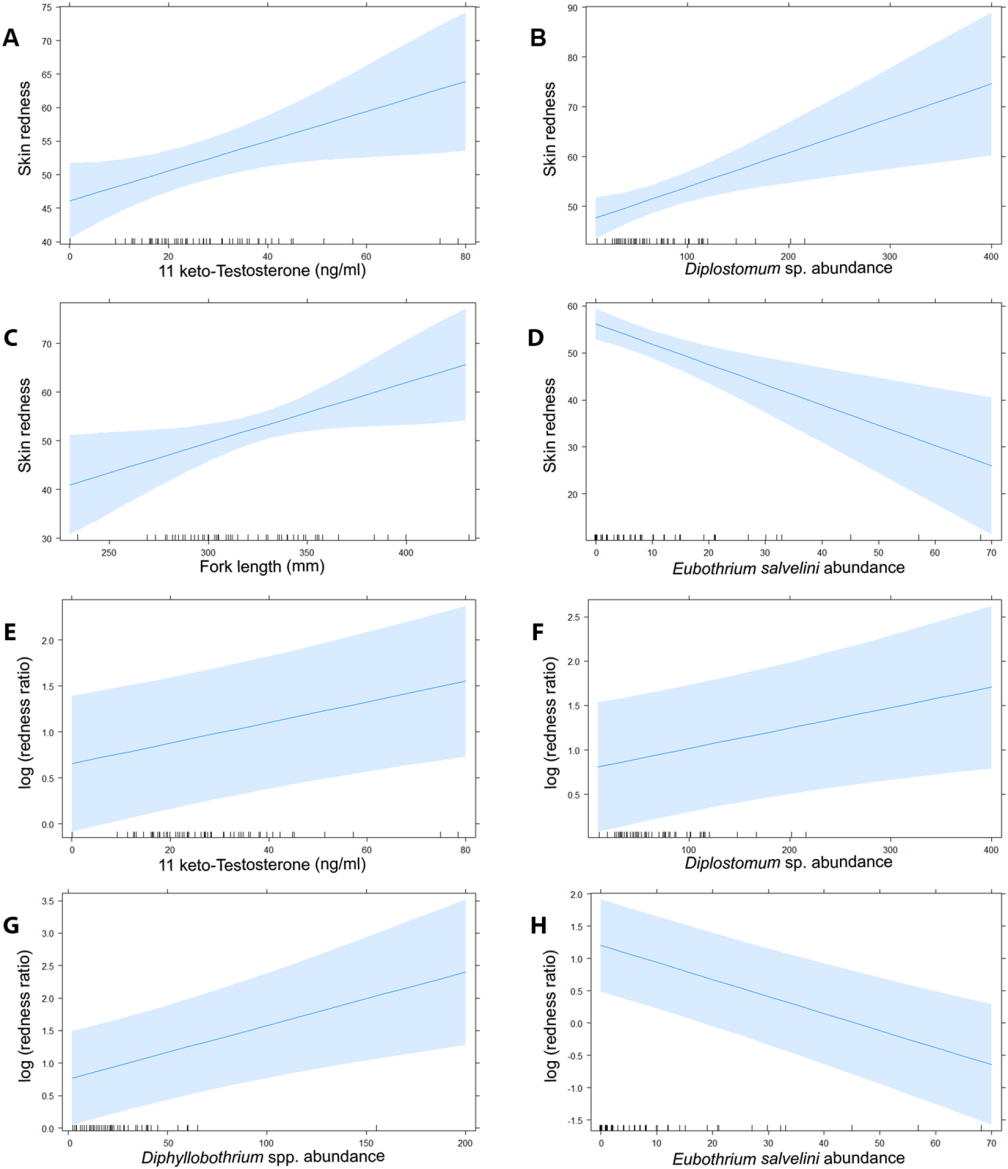
Effects plots illustrating predicted associations between fixed effects and pigmentation in wild-caught male Arctic charr caught in Takvatn, Fjellfrösvatn and Sagelvvatn in Troms County, Northern Norway during spawning season. Predicted associations (significant at *P* < 0.05) between (**A**) 11 keto-Testosterone (ng/ml), (**B**) infection intensity of *Diplostomum* species, (**C**) fish fork length (mm) and (**D**) infection intensity of *Eubothrium salvelini* and skin redness from a linear mixed model (Table 2). Predicted associations (significant at *P* < 0.05) between (**E**) 11 keto-Testosterone (ng/ml), infection intensity of (**F**) *Diplostomum* species, (**G**) *Diphyllobothrium* species and (**H**) *Eubothrium salvelini* and log-transformed ratio between skin and muscle redness (redness ratio) from a linear mixed model (Table 3). Inward ticks on x-axes indicate individual data points and bands indicate 95% confidence.

Final predictors of skin redness following model simplification are listed in Table 2. The total *R*^2^ for the model was 0.49, and the random factor lake accounted for zero variation (*i*.*e*. the model converged to a linear model). Skin redness increased with 11kT plasma concentrations, the abundance of *Diplostomum* parasites and fish length (Fig. 2A–C, Table 2). In contrast, skin redness associated negatively with the abundance of *E*. *salvelini* (Fig. 2D, Table 2). The model included interaction terms indicating that the effect of some parasite species on skin redness depend on levels of co-infection. For instance, the predicted negative association between *E*. *salvelini* and skin redness becomes positive at high intensities (>1000) of *C*. *farionis* (see Supplementary Fig. S3).

Predictors of redness ratios (*i*.*e*. allocation of pigment between skin and muscle) following model selection are listed in Table 3. The *R*^2^ for the model was 0.80 with the random factor lake accounting for 0.49 and fixed factors 0.31. There was a positive association between redness ratios and plasma concentrations of 11kT as well as the abundance of *Diphyllobothrium* spp. and *Diplostomum* sp. (Fig. 2E–G, Table 3). In addition, there was a negative association between redness ratio and the abundance of *E*. *salvelini* (Fig. 2H, Table 3). Interaction terms indicated that this negative association reversed at higher intensities of *Diphyllobothrium* spp. and *C*. *farionis* (see Supplementary Fig. S4).

**Table 3:** Estimated fixed effects (including intercept) predicting redness ratio (log-transformed) after model selection based on AIC values. Marginal *R*^2^ = 0.31, conditional *R*^2^ = 0.80.

Predictor variable	Coefficient (SE)	df	*t* value	*P* value
Intercept	0.96 (0.35)	2.80	2.72	0.078
*C*. *farionis*	0.07 (0.09)	59.84	0.83	0.412
*Diphyllobothrium* spp.	0.18 (0.05)	59.37	3.34	0.001
*Diplostomum* sp.	0.16 (0.07)	60.81	2.47	0.016
*E*. *salvelini*	−0.37 (0.07)	60.38	−5.56	<0.001
11kT	0.17 (0.06)	59.88	2.97	0.004
Fish length	0.14 (0.08)	59.72	1.77	0.082
*C*. *farionis*: *E*. *salvelini*	0.15 (0.04)	59.92	3.66	<0.001
*Diphyllobothrium* spp: *E*. *salvelini*	0.25 (0.09)	58.84	2.97	0.004

## Discussion

The objective of the current study was to test whether parasites benefitting from a more conspicuous host are more abundant in brighter individuals, and vice versa. Using wild-caught naturally infected Artic charr, we found that abundances of parasites awaiting trophic transmission to bird hosts associate positively with skin redness (*Diplostomum* sp.) as well as the ratio of redness in skin versus muscle (*Diplostomum* sp. and *Diphyllobothrium* spp.). We also show that abundance of a parasite species that use charr as definitive host (*E*. *salvelini*) and will gain fitness from hosts investing less in reproduction and secondary sexual characters, is negatively associated with the same phenotypic traits. To our knowledge, this is the first report of contrasting associations between carotenoid-based pigmentation and parasites species at different life history and transmission stages in a teleost host.

Pigmentation varied considerably among individuals in all three study lakes. Intriguingly, this variation in carotenoid pigmentation was predicted by parasite species and also by the sex steroid 11kT (Fig. 2, Tables 2 and 3). In agreement with our findings, Skarstein and Folstad found that the Arctic charr with the brightest red breeding coloration carry more *Diplostomum* eye flukes^1^. A novel aspect of the current study is that redness ratio (ratio of coloration in skin versus flesh) could also be predicted by parasite intensity (*e*.*g*. *Diplostomum*, *Diphyllobothrium*, *and Eubothrium)*. Scoring the redness ratio is a novel approach that better indicates allocation of pigments from internal stores to external signalling than skin redness *per se*.

Since animals do not biosynthesize carotenoids *de novo*, but rely on diet for their supply of carotenoids, positive associations between skin redness and parasite abundance could be explained by dietary intake of carotenoid-rich intermediate host^29^. Put differently, the charr obtains parasites and carotenoids in the same meal. Indeed, the observed trend for a positive association between *C*. *faronis* and red skin coloration observed in the current study (p = 0.06, Table 2) could be ascribed to this phenomenon. This parasite can accumulate in charr over many years, indicating high feeding rates of carotenoid-rich amphipods over the host’s lifespan^30,31^. For *Diphyllobothrium* and *Diplostomum*, on the other hand, it is unlikely that the positive association between the infection intensities of these parasites and carotenoid pigmentation is related to diet. Firstly, *Diphyllobothrium*, transmitted to fish via carotenoid-rich copepods, was a predictor of redness ratio (reflecting allocation of carotenoids), but not of skin redness. Secondly, contrary to all other parasite species investigated in the current study *Diplostomum* sp. is not transmitted to charr via crustaceans (Table 1). Finally, the negative association between copepod-transmitted *E*. *salvelini* and redness/redness ratio in charr further precludes that diet alone can explain positive relationships between parasite intensities and carotenoid coloration.

We also have to consider the possibility that redder individuals (skin redness) or individuals with more pigment allocated to the skin (redness ratio) are more (1) susceptible or (2) exposed to *Diplostomum* sp. The first alternative seems unlikely for two main reasons. Firstly, *Diplostomum* sp. reside in the immune-privileged eye (*i*.*e*. hidden from the immune system) suggesting that the association between this parasite and host color does not involve an immune system response to infection. Like most digenetic trematodes, the free-swimming *Diplostomum* cercariae do however infect the fish by penetrating the skin^32^. Thus, we cannot rule out that penetration and migration of *Diplostomum* in blood vessels^33^ provoke an acute humoral (*i*.*e*. in blood) immune response in the host that limits infection success of the parasite and that individual variation in immune responsiveness could be related to carotenoid-based ornamentation^1^. This would be in line with the immunocompetence handicap-principle suggesting that brightly ornamented individuals signal high quality despite being more susceptible to parasites^1,7^. However, if redder Arctic charr are more susceptible to parasites, we should see positive associations between redness and the total parasite fauna in the animal, which is not the case. In fact, the intestinal cestode *E*. *salvelini* was even negatively associated with skin redness (Fig. 2, Table 2).

Such negative associations between parasite infection intensity and carotenoid ornamentation can reflect a trade-off between use of carotenoids in physiological responses against parasite infection and ornamentation (*i*.*e*. physiological trade-off theory)^5,34^. Such a trade-off could potentially explain the negative association between the copepod transmitted tapeworm *E*. *salvelini* and skin redness in our study since *E*. *salvelini* is a pathogenic parasite in salmonids^35,36^. This assumption is however complicated by the fact that we also see positive associations between *Diphyllobothrium* and redness ratio since this tapeworm is also pathogenic^37^.

In this context, species specific variation in parasite life cycle seems to be a unifying variable that can explain the contrasting associations observed in this study. Thus, if we instead consider the possibility that parasites influence physiological processes involving a redistribution of pigment between muscle and skin, reallocation of pigments available for immune function to external signaling could incur several advantages to parasites awaiting trophic transmission (such as *Diplostomum* and *Diphyllobothrium)*. Firstly, the interior milieu could become more hospitable due to reduced host immune reactivity. Secondly, in the context of parasite-induced trophic transmission (PITT), costly ornaments such as nuptial coloration are associated with increased predation^38–42^. Of note, charr display a striking indifference to human presence during spawning as we and other charr researchers observed (see Supplementary Video kindly donated by Brattli *et al*.^43^) indicating increased risk-taking behavior. Thus positive relationships between parasite abundance and secondary sexual ornamentation could be caused by the presence of parasites that stand to benefit from host reproductive maturation (for instance those awaiting trophic transmission) and their putative influence on host behavior and physiology.

Conversely, parasites that have reached their final, sexually reproducing adult stage (*e*.*g*. *E*. *salvelini*) would instead benefit from a long-lived host, which engages itself in mundane matters such as energy intake rather than energetically costly reproduction associated with potentially risky behavior. In many animals, including Arctic charr, there is often a pronounced cessation or decline in feeding during spawning migrations or other reproductive behaviors (*e*.*g*. courtship, spawning, territoriality, guarding)^44–46^. Clearly, host anorexia in association with spawning would seem disadvantageous for a gamete-producing organism such as *E*. *salvelini*, which inhabits the intestine and pyloric caecae in the charr and feed on intestinal contents. Thus, a negative relationship between parasite abundance and secondary sexual ornamentation could be related to the presence of parasites that do not benefit from neither a conspicuous or anorectic host, let alone both. Of note, *Crepidostomum* sp. and *Proteocephalus* sp., also adult parasites in the intestine, did not show any association to pigmentation.

In short, reproductive maturation brings about coordinated changes in brain and endocrine function (increased steroid levels and associated neurobiological control), behavior (increased risk taking), external appearance (increased conspicuousness), and immune function (reduced), all of which provide potential pathways of influence and benefit to a trophically transmitted parasite. Thus, an efficient route to achieve substantial manipulation of host phenotype with limited intervention would be to interfere with the neuroendocrine system controlling reproduction (*e*.*g*. hypothalamus pituitary gonadal axis, HPG-axis). Intriguingly, the expression of two different secondary sex traits in male sticklebacks, fin size^47^ and carotenoid ornamentation^11^ that are both regulated by sex steroids, positively associate with intensity of parasites awaiting trophic transmission, including *Diplostomum* and *Diphyllobothrium*. Thus, these parasites could, in theory, achieve both these modifications by interfering with the production of sex steroids in their stickleback host.

So far, few reports have demonstrated that parasites can directly alter HPG-axis activity and production of sex steroids in its host. However, in the extensively studied *Toxoplasma gondii* model, where *T*. *gondii* infection in intermediate rodent hosts attenuates innate fear of predator urine, parasite-induced behavioral manipulation requires an increase in testosterone levels^48^. In the current study, accurate analyses of all molecular aspects of endocrine control of the reproductive axis were not feasible because the study animals were stressed during capture. Stress rapidly inhibits the HPG-axis, so any molecular measures could be biased by the capture method if tissue samples are not taken within a few minutes of capture. It is also important to remember that background variation in host physiology cannot be controlled for when using wild-caught fish as study animals. Moreover, steroid levels measured directly following capture does not necessarily reflect historic steroid production and exposure. Nonetheless, in this study the sex steroid 11kT (which is the primary end-point in the male HPG-axis in salmonids - as opposed to testosterone in mammals) positively associated with redness ratio. This observation supports that somatic redistribution of carotenoids is mediated by 11kT in Arctic charr^49^. In summary, it would appear that the individuals that are most heavily infected with parasites awaiting trophic transmission also demonstrate the strongest investment in reproduction.

In conclusion, our findings may represent the first empirical data demonstrating contrasting associations between carotenoid coloration and parasite infection intensities related to parasite species and life cycle. In short, parasites benefitting from a more conspicuous host are more abundant in brighter individuals, and vice versa. Hence, to fully explain the relationship between carotenoid pigmentation and parasitism, it is necessary to consider the wider range of parasites species and associated life histories occurring within single hosts. Moreover, the significant interaction terms (Supplementary Figs 3 and 4) in our models show that the association between parasite intensity and pigmentation depend on levels of co-infection. This substantiates the complex nature of parasite infections that can be observed in wild populations as opposed to in controlled experimental settings. Nonetheless, proximate mechanisms underlying the observed contrasting associations between parasitism and coloration should be addressed in experimental studies where background variation in host physiology, behavior, timing and magnitude of infections etc. can be controlled. The direction of the association should in this context be analysed with respect to expected fitness outcome for specific parasite species and life stages. We hope that the present study can encourage taking on a parasite’s perspective on host carotenoid allocation also in other model systems where sexual ornamentation is reported to associate with parasite abundance.

## Supplementary information

Dataset

Supplementary video 1

Dataset 1

## Data Availability

All relevant data are within the manuscript and its Supplementary Material.
