# Supplementary material for: Contrasting associations between breeding coloration and parasitism of male Arctic charr relate to parasite species and life cycle stage
Source: Sci Rep. 2019 Jul 23;9:10679. doi: 10.1038/s41598-019-47083-x (PMC6650492; doi:10.1038/s41598-019-47083-x)
Supplement: Supplementary file 1 — Dataset [file 41598_2019_47083_MOESM1_ESM.pdf]

Contrasting associations between breeding coloration and parasitism of male Arctic charr relate to parasite species and life cycle stage.

Johansen, I.B., Henriksen, E. H., Shaw, J.C., Mayer, I., Amundsen, P. A. and Øverli, Ø.

Supplementary Material

Supplementary Tables

Table S1. Characteristics of the three study lakes. Fish species abbreviations: AC = Arctic charr, BT = brown trout, TS = three-spined stickleback.

	Fjellfrøsvatn	Takvatn	Sagelvvatn
Altitude (m a.s.l.)	125	214	91
Area (km^2)	5.5	14.2	5.0
Max. depth (m)	80	80	80
Fish community	AC, BT	AC, BT, TS	AC, BT, TS

Table S2. Mean length, skin redness and parasite infections for Arctic charr sampled in the three study lakes. P = parasite prevalence in per cent, MA = mean abundance (SE). Only 25 fish in Takvatn were screened for *C. farionis*.

	**Fjellfrösvatn**	**Sagelvvatn**	**Takvatn**
N Arctic charr	14	23	26
Arctic charr mean length in mm (SE)	310.4 (9.4)	296.7 (3.6)	348.9 (6.3)
Skin redness (SE)	58.6 (5.2)	51.1 (2.4)	54.8 (2.9)
	**P, MA (SE)**	**P, MA (SE)**	**P, MA (SE)**
Crepidostomum sp.	100, 83(32)	13, 2 (2)	92, 12 (3)
Cyathocephalus truncatus	64, 4(1)	0, -	4, 0.04 (0.04)
Cystidicola farionis	93, 226(41)	0, -	96, 467(78)
Diphyllobothrium spp.	100, 31(4)	100, 44(8)	100, 18(3)

Diplostomum sp.	100, 158(29)	100, 60(5)	100, 51(6)
Eubothrium salvelini	100, 11(3)	30, 2(1)	96, 15(4)
Proteocephalus sp.	7, 1(1)	100, 275(58)	77, 25(7)

Supplemetary Figures and Figure legends

Figure S1

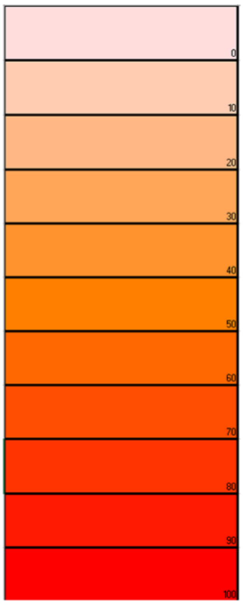

Figure S1. Red scale for colour determination of skin and muscle.

Figure S2

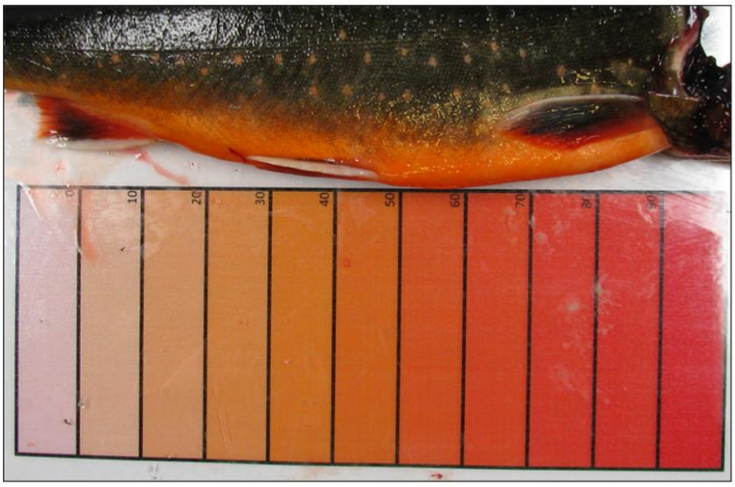

Figure S2. Example of skin colour determination using the red scale. Arctic charr in picture was scored with a skin redness of 55.

Figure S3

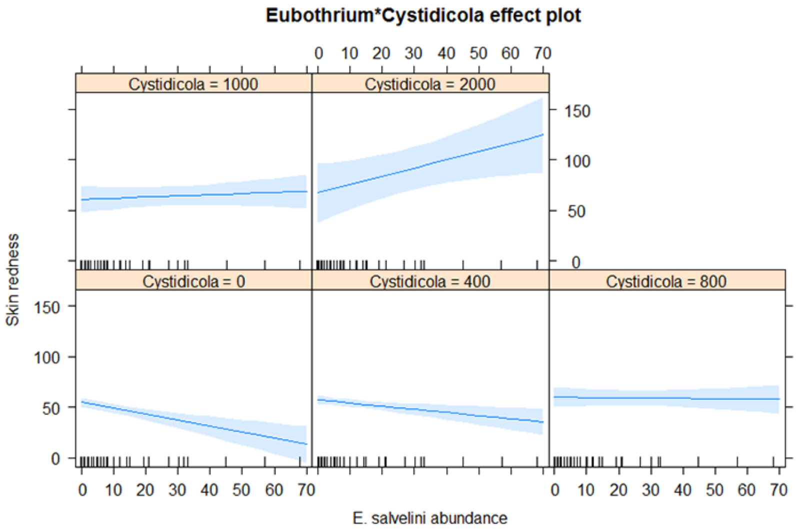

Figure S3. Predicted association between *Eubothrium salvelini* abundance (x-axis) and skin redness (y-axis) from linear mixed model at five different abundances (0, 400, 800, 1000, 2000) of *Cystidicola farionis*.

Figure S4

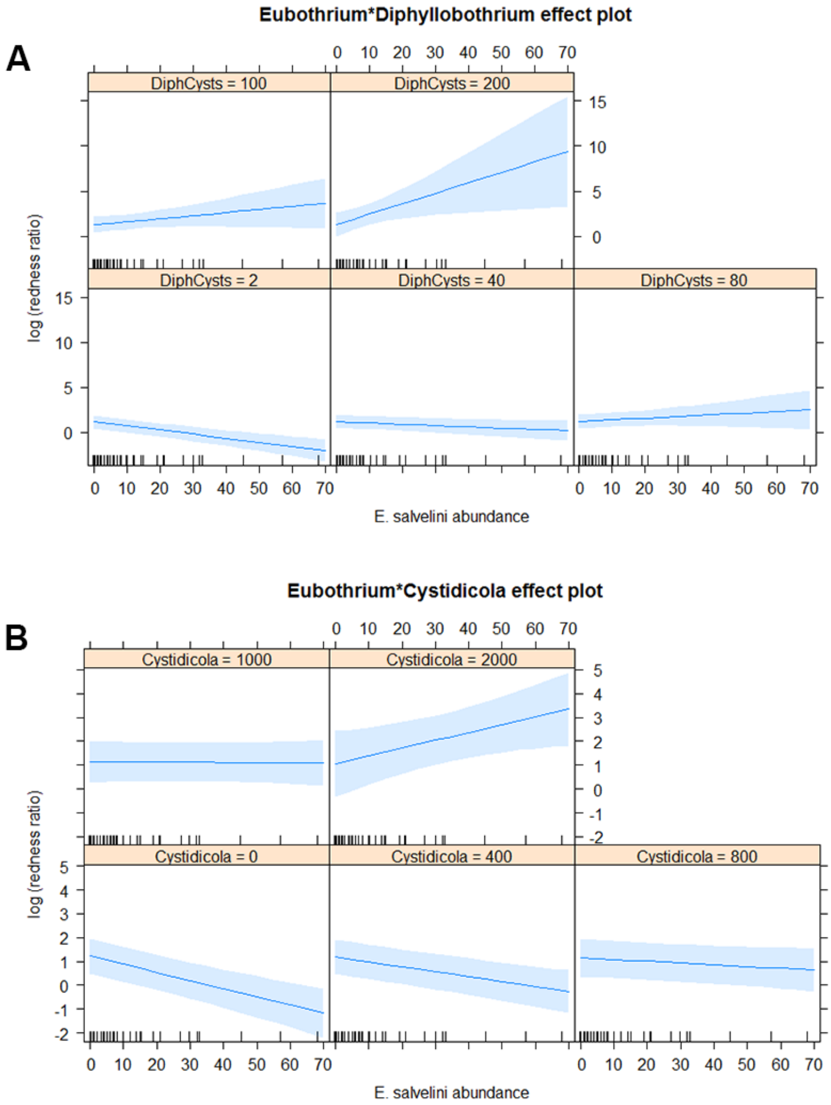

Figure S4. Predicted association between *Eubothrium salvelini* abundance (x-axis) and redness ratio (y-axis, log-transformed) from linear mixed model at five different abundances of *Diphyllobothrium* spp. (A) and *Cystidicola farionis* (B).
